# A Review about Regulatory Status and Recent Patents of Pharmaceutical Co-Crystals

**DOI:** 10.15171/apb.2018.042

**Published:** 2018-08-29

**Authors:** Arun Kumar, Sandeep Kumar, Arun Nanda

**Affiliations:** Department of Pharmaceutical Sciences, Maharshi Dayanand University, Rohtak-124001, India.

**Keywords:** Pharmaceutical Co-crystals, Crystal Engineering, Regulatory guidelines, Patents

## Abstract

Pharmaceutical Co-crystals are not new, they have gained much attention since the last decade among scientists and pharmaceutical industry. Pharmaceutical co-crystals are multicomponent systems composed of two or more molecules and held together by non-covalent interactions. The development of pharmaceutical co-crystals, a new solid crystalline form, offer superior physico-chemical properties (such as melting point, stability, solubility, permeability, bioavailability, taste masking, etc.) without altering the pharmacological properties. Recently, with the upsurge in the growth of Pharmaceutical co-crystals, the major concern is over the regulatory status of co-crystals. With the new guidelines from United States Food and Drug Administration (USFDA) and European Medicines Agency (EMA), the status has become even more complicated due to significantly different opinions. This review highlights whether co-crystals fulfil the requirements for the grant of a patent or not and how cocrystals are going to affect the present scenario of pharmaceuticals.

## Introduction

Pharmaceutical co-crystals have established a new paradigm in the solid-state modification. The formation of API co-crystal offers a wide range of physical and chemical enhancements to the properties of drugs without altering their chemical nature, thereby maintaining its pharmaceutical importance as such.^1^ This is evident from the fact that regulatory bodies like United States Food and Drug Administration (USFDA) and European Medicines Agency (EMA) have published regulatory guidelines to clarify the status of co-crystals in their respective regions. Pharmaceutical and Biotechnology companies rely upon intellectual protection for safeguarding their products. In order to maintain revenues generated through these products as a means to recover the resources and money spent on research and development, the presence of proper regulatory guidelines is expected to significantly affect the development and quality control as well as intellectual properties aspects of pharmaceutical cocrystals and their formulations.^2^

However, the concern that remains unanswered is whether the standard development and manufacturing processes that were initially designed for salt-based formulations can also be used for co-crystal based formulations in order to achieve the desired product quality that is required to ensure the safety and efficacy.^3^ Moreover, from regulatory perspective the addition of another component to the drug formulation could mandate additional bioequivalence, clinical and toxicity studies.

This article focuses on listing recent developments regarding regulatory status of co-crystals in different regulatory regions, the effect of these regulatory guidelines and intellectual protection in the field of crystal engineering. Another point to be probed is whether co-crystals are eligible for patent protection or not as per the literature and guidelines available on pharmaceutically acceptable co-crystals.

## Pharmaceutical Co-Crystals

Poor solubility has been a crucial issue in the development of a pharmaceutical dosage form. Amorphous solids may be considered as a good choice but these solids have their own limitations related to stability.^4^ The composition and the arrangement of molecules/ions in a crystal lattice directly affects the crystal properties, it means that exerting control over the composition by selecting a co-former from a wide range can lead to co-crystals of desired physicochemical properties. This was the reason crystal engineering gained impetus in pharmaceuticals for the enhancement of stability/solubility of pharmaceutical formulations.^5^ Co-crystals can be made for both complex drugs containing sensitive functional groups as well as for drugs containing non-ionizable moieties and that is the unique advantage of co-crystals over salts. The other key advantages of co-crystals are that co-former modifies only the physicochemical properties of drug without altering the molecular structure and pharmacological properties of the drug.^6^

Cocrystals are a long known but understudied class of crystalline solids. In 1844, Wohler was the first to obtain a co-crystal of the 1:1 ratio between Benzoquinone and Hydroquinone (Quinhydrone).^7^ Desiraju in 1989 defined crystal engineering as “the understanding of intermolecular interactions in the context of crystal packing and in the utilization of such understanding in the design of new solids with desired physical and chemical properties”.^8^ However, pharmaceutical co-crystals have attracted interest from scientists in the past decade and now much of work has been done in this field, mainly because co-crystallization utilizes non-covalent interactions and supramolecular synthons to control the organisation of molecules inside the crystal lattice, co-crystals possess better thermodynamic stability, purity and processing characteristics over amorphous solids.^9^

In 2004, Almarsson and Zaworotko proposed the least controversial definition of co-crystals as “co-crystals are those that are formed between an active pharmaceutical ingredient (API) and a co-former also called as crystal former (CF), which under ambient conditions are solids. This definition is not limited to two components, that the co-crystal can be multi-component”. The components in the co-crystal interact by hydrogen bonding or other non-ionic and non-covalent interactions such as halogen or π-π interactions.^10^ In 2011, a bilateral meeting jointly sponsored by the Indo−U.S. Science and Technology Forum (IUSSTF) was held on Pharmaceutical Cocrystals and Polymorphs where meeting a generally accepted definition of co-crystals was evolved which reads as follows ‘‘Cocrystals are solids that are crystalline single-phase materials composed of two or more different molecular and/or ionic compounds generally in a stoichiometric ratio which are neither solvates nor simple salts’’.^11^ USFDA defined that cocrystals are crystalline materials which are composed of two or more molecules in the same crystalline lattice and associated by non-ionic and non-covalent bonds.^12,13^ Pharmaceutical co-crystals belong to a subclass of co-crystals wherein one of the components is a biologically active substance (an API) while the other one the co-former is drug or food grade substance (generally regarded as safe). Inside the crystal lattice the two components interact through non-covalent interactions such as hydrogen bonding in fixed stoichiometric ratio.^14^

The foundation of crystal engineering lies in the concept of supramolecular chemistry. The basic tenet of supramolecular chemistry is the molecular recognition between complementary molecular fragments giving rise to self-organization of molecules to give a supramolecular function.^15^ Co-crystallization has been shown to significantly modify the physicochemical properties of drug substances such as the, permeability, bioavailability, solubility and dissolution rate, compaction and tableting, physical form, biochemical and hydration stability, melting point, etc.^6,14,16-18^

Selection of suitable co-formers and screening of co-crystals for a drug are the main challenges to overcome during the process of cocrystal development. Selection of co-formers is mainly done by researchers using theoretical or experimental approaches. Different approaches i.e. hydrogen bonding propensity, Cambridge Structure Database, supramolecular synthons,^18^ ∆pKa values,^19^ Fabine’s method,^20^ COSMO-RS screening,^21^ Hansen solubility parameters,^22^ virtual cocrystal screening,^23^ thermal methods (including DSC screening,^24^ hot stage microscopy^25^ and saturation method^26,27^) and others methods are reported in the literature by the scientists for the selection of the appropriate co-former for a drug and screening of cocrystals.

Academicians and scientists reported various methods (such as solution based, grinding, and other advanced methods freeze drying, spray drying, hot melt extrusion, supercritical carbon dioxide processing, ultrasound crystallization and microfluidic jet dispersion) for the synthesis of cocrystals with their pros and cons.^14,28-31^ Bavishi and Borkhataria described the “Spring and Parachute” concept for better understanding in the improvement of solubility and dissolution rate of drug.^32^ Different characterization techniques such as structural analysis (crystallographic studies, Hirshfeld surface analysis and spectroscopic characterization), thermal analysis (Differential scanning calorimetry, thermogravimetric analysis and hot stage microscopy) and pharmaceutical characterizations (solubility and dissolution profile, stability, bioavailability and pharmacokinetic studies) have been used for determining the successful synthesis and pharmaceutical utility of cocrystals.^14,18,33^

A co-crystal is also possible between two biologically active molecules that is drug: drug co-crystal. The motive behind multidrug cocrystals is towards developing combination therapies, prevention of multi-drug resistance, synergistically increasing the action of drugs, reducing side effects, etc.^34^ Bhatt *et al.*, reported a co-crystal between Lamivudine and Zidovudine (both anti-viral drugs active against HIV).^35^

## Regulatory prospects and patentability issues of Co-Crystals

Once a pharmaceutical cocrystal with promising results is developed, the next step would be gaining regulatory approval so that it can be brought to market. However, the lack of clear regulatory guidelines is a major issue to tackle with. Over the last decade, cocrystal development has seen enormous growth, there are even few patents granted for cocrystals. For an invention, in order to be patentable, the invention must fulfil the three conditions such as novelty, non-obviousness and utility or usefulness.^36,37^

## Novelty

Desiraju in his book “Pharmaceutical salts and co-crystals: retrospect and prospects” mentioned that pharmaceutical co-crystals are new composition of matter and hence should satisfy the requirement of novelty for the grant of patent.^38^ Andrew Trask in his article titled "An Overview of Pharmaceutical Cocrystals as Intellectual Property" also stated that pharmaceutical co-crystals should satisfy the novelty condition as equally as salts. Both Desiraju and Andrew emphasised that since co-former screening is a daunting work and co-formers are selected from a huge official list of GRAS compounds and the result of co-crystallization is not easily predictable, co-crystals may or may not be formed. Apart from this, the properties of the synthesized co-crystals cannot be anticipated. But the situation is completely different, FDA didn't even consider co-crystals in the same class as that of salts or polymorphs.^39^

## Non-obviousness

Non-obviousness means that if someone skilled in the relevant field of technology and familiar with its subject matter invented it with comparative ease; such an “invention” would be novel but obvious to that person**.** Desiraju described that unlike salt formation wherein an acid is necessary to form a salt with a base, the identification of a co-former is hardly an ever routine.^38^ According to Trask, in spite of a number of co-crystals screening methods available, there is no confirmed way to predict whether two molecules will form a hydrogen bond and a co-crystal will be formed. There are a lot of factors that govern the co-crystallization process and still there is a need to better understanding of this process. Moreover, co-crystal structure cannot be predicted from the available sources. Hence co-crystals well satisfy the Non-obviousness criteria too.^39^

## Utility

In case of Pharmaceutical co-crystals, the only criteria that needs to be demonstrated in order to obtain a patent is utility or application of the invention. Co-crystals offer opportunities similar to that of polymorphs. They are clearly new substances, problems of inherent anticipation are not likely to arise so often and more of them can be made for any given API, expanding the pharmaceutical space around it and consequently the types of advantageous properties that may be accessed.^38^ As per Trask, co-crystal of an API shares the same patentable therapeutic utility as its parent API. The enormous research on co-crystals in the past decade indicates that co-crystals offer vast opportunities for enhancement of the properties of an API, which in turn increases its utility and hence also the chances of patentability.^39^

## Regulatory Perspectives

USFDA was the first regulatory body to publish guidelines for pharmaceutical co-crystals in 2013; the guidance classified Pharmaceutical co-crystals as drug product intermediate and treated them similar to API-excipient molecular complexes. Further the document stated that:

- The API and the co-former should exist in neutral states and interaction among them should be non-covalent/non-ionic.
- The value of ΔpKa should be less than 1 that is ΔpKa [pKa (base) - pKa (acid)] < 1.
- The API and the co-former should completely dissociate before reaching the site of pharmacological activity.^12^

The revised guidelines of FDA published in 2016, classify the pharmaceutical co-crystals as a special case of solvates and hydrates and placed pharmaceutical co-crystals in the regulatory classification similar to that of a polymorph of the API. Additionally, FDA required an *in-vitro* evaluation based on dissolution and/or solubility is generally considered sufficient to demonstrate that the active drug dissociates completely from the co-former.^13^

EMA's opinion on Pharmaceutical co-crystals differs considerably from that of FDA. EMA published a paper in 2014 about cocrystals and placed co-crystals in the same class as that of salts. The regulations also classify that co-crystals are eligible for generic application in the same way as salts. For a co-crystal to be considered as New Active Substance status (NAS), the co-crystals should demonstrate the difference in efficacy and/or safety with respect to that of API. NAS status for other routes of administration will be dependent on the therapeutic moiety that is present at the site of pharmacological action when compared to that of the authorised product.^40^ The USFDA and EMA classification of Pharmaceutical co-crystals is summarised in Table 1.

## Patents on Co-Crystals: Case Studies

Over the past decade, Pharmaceutical co-crystals have seen enormous growth and a large number of research papers and patents have been filed all over the world and till date, a number of patents related to co-crystals and multi-drug co-crystals have been approved. Some of the recently approved pharmaceutical co-crystal formulations and list of approved patents on pharmaceutical co-crystals in USA, Europe, International (worldwide) and multi-drug co-crystals patents have been enlisted in the Table 2, Table 3, Table 4 and Table 5 respectively.

## Entresto

The US Food and Drug Administration (FDA) on July 7, 2015, approved a multidrug co-crystal formulation of sacubitril and valsartan (brand name Entresto, Novartis) to reduce the risk for cardiovascular and chronic heart failure. Entresto was a new oral combination approved through fast-track review.^41^

## Lexapro

Lexapro is a co-crystal formulation composed of escitalopram and was approved in 2009 under the brand name Lexapro, for the treatment of major depressive and anxiety disorders.^42^

## Steglatro

The Food and Drug Administration (USFDA) has approved Ertugliflozin co-crystal formulation (Ertugliflozin cocrystal with 5-oxo-proline) under the brand name Steglatro™.^43,44^

## Suglat® (Ipragliflozin: L-proline)

An Ipragliflozin: L-Proline co-crystal of the molecular ratio 1:1 was developed by Astellas Pharma and Kotobuki Pharmaceuticals, Ipragliflozin is a sodium-glucose co-transporter-2 (SGLT2) inhibitor. The co-crystal formulation was approved and is available under the trade name Suglat® in Japan.^45,46^

## TAK-020—Gentisic acid Co-crystals

Takeda pharmaceuticals developed a new co-crystal-based formulation named TAK-020 developed for the treatment of rheumatoid arthritis (Bruton’s tyrosine kinase inhibitor). The co-crystal has completed phase-I clinical trials.^46^

**Table 1:** Comparison between United States Food & Drug Administration and European Medicines Agency guidelines^3^

**Regulatory considerations**	**Food & Drug Administration guidance (2013 & 2016)**	**European Medicines Agency reflection paper (2015)**
**Regulatory category**	Polymorph of the Active Pharmaceutical Ingredient	Active Pharmaceutical Ingredient
**Composition**	Active Pharmaceutical Ingredient & a food or drug grade co-former	Active Pharmaceutical Ingredient and co-former in fixed stoichiometric ratio
**Interaction in crystal**	Non-ionic/non-covalent interactions	Non-ionic/non-covalent interactions
**Co-former role**	Excipient	Reagent
**New Chemical Entity /New Active Substance Registration**	No	Possible if shown difference in efficacy/safety
**Similarity with Active Pharmaceutical Ingredient**	Similar	Similar unless demonstrated different efficacy/safety
**Classification**	Polymorph of Active Pharmaceutical Ingredient	Salts of Active Pharmaceutical Ingredient
**Cocrystal and salt**	Differences in interaction and regulatory pathways	Regulation dependent on efficacy/safety
**Drug Master File/Active Substance Master File requirement**	No	Required for New Active Substance registration

## Aripiprazole

Aripiprazole is a co-crystal formulation available in the market under the brand name Abilify®, Abilify consists of co-crystals comprising aripiprazole and fumaric acid. Aripiprazole is a psychotropic drug useful for the treatment of schizophrenia.^47^

## Tramadol-Celecoxib (1:1) Cocrystal

E-58425 comprising celecoxib and tramadol (1:1) was developed by Enantia and Esteve, R&D, Spain, and patented by Laboratorios Del. This is an example of multidrug cocrystal, which is under clinical development. Synergistic action between its components will help to achieve the therapeutic benefits at lower and tolerable doses of each component. A phase II proof-of-concept study in acute postoperative pain has shown that the cocrystals demonstrated superior efficacy and safety over both placebo and a standard. The co-crystal based formulation is currently in phase-III of clinical trials.^48-50^ Some other patents about the novel cocrystals of tiotropium bromide and ticagrelor drugs have also been granted to Boehringer Ingelheim Pharma Gmbh Co and Astrazeneca respectively.^51^

**Table 2:** Composition patents issued in the USA for pharmaceutical cocrystals^2^

**US Patent No.**	**Date of issue**	**Assignee**	**Compound(s)**	**Ref.**
US6001996	14 Dec, 1999	Eli Lilly & Co., Inc.	Complexes of (carba)cephalosporins with parabens	52
US7446107	4 Nov, 2008	TransForm Pharmaceuticals, Inc.	Itraconazole; cocrystals with acarboxylic acid	53
US7625910	1 Dec, 2009	Astra Zeneca AB	AZD1152; a phosphate prodrug and maleic acid cocrystal	54
US8097592	17 Jan, 2012	Astellas Pharma Inc., Kotobuki Pharmaceutical Co. Ltd.	SGLT-2 Inhibitor, l-proline cocrystal	55
US8124603	28 Feb, 2012	Thar Pharmaceutical	Meloxicam with various carboxylic acids, aliphatic and aromatic, and maltol and ethyl maltol	56
US8163790	24 Apr, 2012	New Form Pharmaceuticals, Inc.	Metronidazole cocrystals with gentisic acid and gallic acid (specific x-ray reflections in each case) and a cocrystal of imipramine HCl and (+)-camphoric acid	57
US20170044176 A1	16 Feb, 2017	Euticals Spa	Cocrystal of tiotropium bromide and lactose monohydrate	58
US20170224724 A1	10 Aug, 2017	University Of South Florida	Co-crystal (ICC) of lithium with salicylic acid and 1-proline	59
US20170101433 A1	13 Apr, 2017	Amri Sci. Llc.	Co-crystal of progesterone and a co-former selected from the group consisting of vanillic acid, benzoic acid, salicylic acid, cinnamic acid, and vanillin.	60

**Table 3:** European patents on Co-Crystals^2^

**Patent no.**	**Date of issue**	**Assignee**	**Componds**	**Ref.**
EP1755388B1	6 Oct, 2010	TransForm Pharmaceuticals, Inc.	Mixed cocrystals of modafinil	61
EP2185546B1	26 Oct, 2011	Vertex Pharmaceuticals, Inc.	Cocrystals and pharmaceutical compositions, telaprevir (VX-950)	62
EP2334687B1	4 Jan, 2012	Pfizer Inc.	SGLT-2 inhibitors, l-proline and pyroglutamic acid cocrystals	63
EP2300472B1	18 Jan, 2012	Boehringer Ingelheim Intl. GmBH	Glucocorticoid analogs, phosphoric acid and acetic acid cocrystals	64
EP2114924B1	25 Jan, 2012	Vertex Pharmaceuticals Inc.	Cocrystals of telaprevir with 4-hydroxybenzoic acid; solvates	65
EP2288606B1	15 Feb, 2012	Bayer Pharma Ag	Rivaroxaban cocrystal with malonic acid	66
EP1608339B1	21 Mar, 2012	McNeil PPC	Celecoxib cocrystal with nicotinamide	67
EP3210975 A1	30 Aug, 2017	Enantia, S.L.	Cocrystals of Lorcaserin hydrochloride and an organic diacid	68
EP3240575 A1	8 Nov, 2017	Dr. Reddy's Laboratories Ltd.	co-crystal of carfilzomib with maleic acid	69

**Table 4:** International patents on Co-Crystals

**Patent no.**	**Date of issue**	**Assignee**	**Compounds**	**Ref.**
WO2017191539 A1	9 Nov, 2017	Aurobindo Pharma Limited	dl-proline co-crystal of dapagliflozin	70
WO2017172811 A1	5 Oct, 2017	Intra-Cellular Therapies, Inc.	Co-crystal forms of 1-(4-fluoro-phenyl)-4-((6bR,10aS)-3-methyl-2,3,6b,9,10,10a-hexahydro-1H,7H-pyrido[3´ 4´:4,5]pyrrolo[1,2,3-de]quinoxalin-8-yl)-butan-1-one and isonicotinamide and nicotinamide.	71
WO2017144598 A1	31 Aug, 2017	Enantia, S.L.	Cocrystals of Lorcaserin hydrochloride and an organic diacid	72
WO2017115284 A1	6 Jul 2017	Leiutis Pharmaceuticals Pvt, Ltd.	Adipic acid co-crystal of Agomelatine	73
WO2016156127 A1	6 Oct 2016	Ratiopharm Gmbh	Co-crystal of ibrutinib and carboxylic acid	74

**Table 5:** Patents on multi-drug Co-Crystals^34^

**Drug combination**	**Therapeutic category**	**Refs.**
ASA–theanine	NSAID and psychoactive	75
Cyprodinil–dithianon	Fungicides	76
Ciprofloxacin and norfloxacin with various co-crystal formers	Antibacterial	77
Mesalamine with alpha amino acids, flavones, and nutraceuticals	Anti-inflammatory	78
Metformin–oleoylethanolamide	Antidiabetic and anti-obesity	79
Quercetin–metformin	Antioxidant and antidiabetic	80

## Co-crystals and Evergreening of Patents

Ever-Greening and follow on patent are used to refer the patents that are filled to protect the additional aspects of further improvements to an invention. This provision of follow on patents to an existing invention was included in law so as to encourage further research as a means to obtain pharmaceutical products that are much safer and effective. While the terms “ever-greening patent” and “follow-on patent" are both used to refer to patents that protect pharmaceutical formulations, new forms of active agents, processes for manufacturing active agents, new uses for pharmaceutical products, new combinations of active agents, new dosing regimens, most of the pharmaceutical companies have ill practiced in this provision and have created picket fences of minor improvements filled over the parent patent and hence successfully thwarting any generic entry in the market and maintaining their monopoly for extended periods of time. While, Co-crystallisation is an approach that results in drug products that seem to satisfy the conditions of novelty, non-obviousness and utility but it may definitely stimulate investigation of older APIs for new benefits and this may in turn lead to ever greening of existing drug patents.^81^

## Conclusion

Co-crystallization is a flourishing approach with direct application to the pharmaceutical industry. It is quite evident from the amount of interest shown by both academia and pharmaceutical industry that in near future pharmaceutical cocrystals will be one of the viable and important solid forms of pharmaceuticals that should be available in the market. The value of co-crystals to the pharmaceutical industry should become clearer, particularly with respect to several relevant legal and regulatory issues, as products containing cocrystal technology emerge from pharmaceutical development pipelines into the market. It will also lead to screening of older API’s to see new benefits and improvements of existing drugs. Co-crystal formation offers tremendous scope for controlled modification of the key pharmaceutical properties such as dissolution rate, solubility, compressibility, melting point, stability, bioavailability and permeability. There is a need to explore into an understanding of cocrystallization mechanism, *in-vivo* behaviour of cocrystal for better therapeutics and other unanswered questions like polymorphic transformation, the concepts of supramolecular synthesis, and crystal engineering remain largely underexploited. Pharmaceutical cocrystals generally appear patentable when measured against the criteria of novelty, utility, and non-obviousness as evident from the fact that number of patents filed throughout the world by various pharmaceutical industries and research groups are also increasing at a fast pace. The challenges that lie ahead include scaling up the production of the pharmaceutical cocrystals, preceded by discovery of new scale up methods, and high throughput screening of the possible co-crystal with various co-formers and their polymorphs.

## Acknowledgments

Authors thank Maharshi Dayanand University, Rohtak and University Grant Commission, New Delhi for providing University Research Fellowship and Basic Scientific Research Fellowship respectively, for working in the project.

## Ethical Issues

Not applicable.

## Conflict of Interest

The authors declare no conflict of interests.
